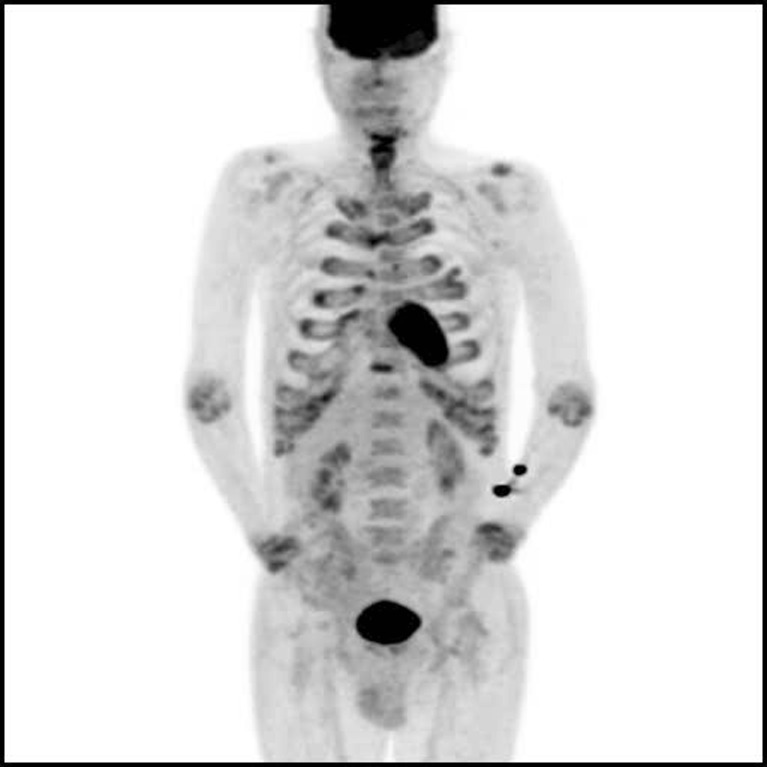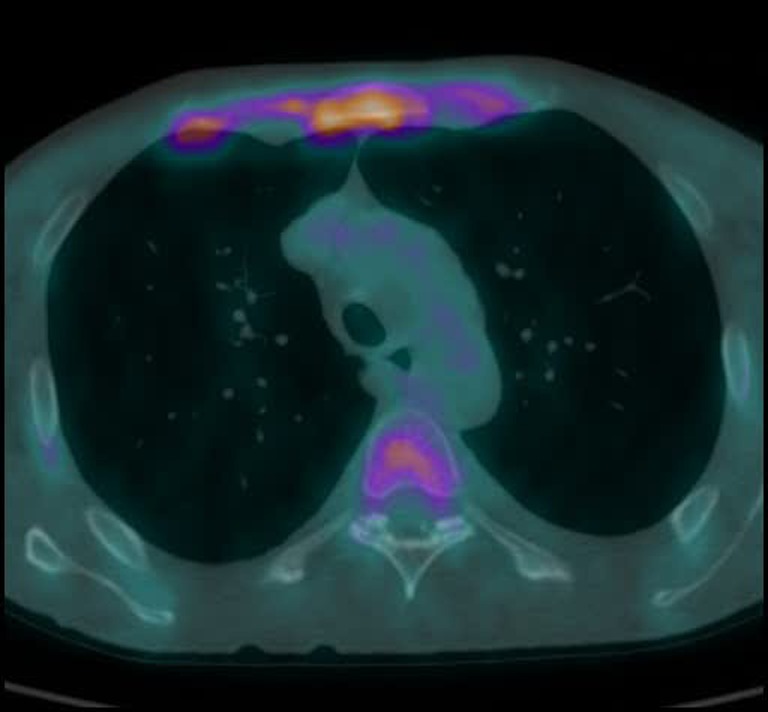# Relapsing polychondritis detected in PET/CT

**DOI:** 10.1007/s00259-012-2128-6

**Published:** 2012-04-20

**Authors:** Rafał Czepczyński, Izabela Guzikowska-Ruszkowska, Anna Wyszomirska

**Affiliations:** 1 Department of PET/CT, Euromedic Diagnostics, Poznań, Poland; 2 Department of Endocrinology and Metabolism, Poznan University of Medical Sciences, Poznań, Poland; 3 Department of General Radiology, Poznan University of Medical Sciences, Poznań, Poland; 4 Department of Endocrinology, Poznan University of Medical Sciences, Przybyszewskiego 49, PL-60-355 Poznań, Poland

We report a case of relapsing polychondritis (RP) in a patient in whom PET/CT using ^18^F-FDG performed due to a suspicion of malignancy led to the diagnosis of RP.

A previously healthy man, aged 57 years, was admitted to hospital for the diagnosis of chronic fever and weight loss of about 5 kg. Extensive laboratory and imaging evaluation showed only elevated C-reactive protein levels up to 180 mg/l (normal value <10 mg/l) and erythrocyte sedimentation rate up to 118 mm/h. Endoscopic evaluation showed signs of chronic laryngitis and sinusitis, oesophageal candidiasis, chronic gastritis and a benign sigmoid polyp. Antibacterial and antifungal therapy did not cause any significant improvement in the fever or laboratory findings.

PET/CT using ^18^F-FDG showed diffuse ^18^F-FDG accumulation in all costal cartilages and in the sternal angle (SUV_max_ = 4.5). Additionally, symmetrically increased ^18^F-FDG activity in the joints of the upper extremities (elbows and wrists) was found. A moderately increased ^18^F-FDG concentration in the hypopharynx was also visible. The CT scan demonstrated thickened tracheal mucosa that did not show any ^18^F-FDG activity. The characteristic complex of signs and symptoms after exclusion of malignancy led to the diagnosis of RP – a rare autoimmune disease characterized by inflammation of cartilaginous tissues of the larynx, trachea and ears [1–3].

The patient was treated with glucocorticosteroids that provided rapid improvement of the symptoms and laboratory parameters.